# Scattering of therapeutic radiation in the presence of craniofacial bone reconstruction materials

**DOI:** 10.1002/acm2.12776

**Published:** 2019-11-29

**Authors:** Joonas Toivonen, Mikko Björkqvist, Heikki Minn, Pekka K. Vallittu, Jami Rekola

**Affiliations:** ^1^ Department of Otorhinolaryngology ‐ Head and Neck Surgery Turku University Hospital Turku Finland; ^2^ Department of Biomaterials Science Institute of Dentistry and Turku Clinical Biomaterials Centre ‐ TCBC University of Turku Turku Finland; ^3^ Department of Medical Physics Division of Medical Imaging Turku University Hospital Turku Finland; ^4^ Department of Oncology and Radiotherapy Turku University Hospital and University of Turku Turku Finland; ^5^ City of Turku Welfare Division Turku Finland

**Keywords:** bioglass, craniofacial bone reconstruction, fiber reinforced composite, radiation scattering, radiotherapy

## Abstract

**Purpose:**

Radiation scattering from bone reconstruction materials can cause problems from prolonged healing to osteoradionecrosis. Glass fiber reinforced composite (FRC) has been introduced for bone reconstruction in craniofacial surgery but the effects during radiotherapy have not been previously studied. The purpose of this study was to compare the attenuation and back scatter caused by different reconstruction materials during radiotherapy, especially FRC with bioactive glass (BG) and titanium.

**Methods:**

The effect of five different bone reconstruction materials on the surrounding tissue during radiotherapy was measured. The materials tested were titanium, glass FRC with and without BG, polyether ether ketone (PEEK) and bone. The samples were irradiated with 6 MV and 10 MV photon beams. Measurements of backscattering and dose changes behind the sample were made with radiochromic film and diamond detector dosimetry.

**Results:**

An 18% dose enhancement was measured with a radiochromic film on the entrance side of irradiation for titanium with 6 MV energy while PEEK and FRC caused an enhancement of 10% and 4%, respectively. FRC‐BG did not cause any measurable enhancement. The change in dose immediately behind the sample was also greatest with titanium (15% reduction) compared with the other materials (0–1% enhancement). The trend is similar with diamond detector measurements, titanium caused a dose enhancement of up to 4% with a 1 mm sample and a reduction of 8.5% with 6 MV energy whereas FRC, FRC‐BG, PEEK or bone only caused a maximum dose reduction of 2.2%.

**Conclusions:**

Glass fiber reinforced composite causes less interaction with radiation than titanium during radiotherapy and could provide a better healing environment after bone reconstruction.

## 1 INTRODUCTION

Treatment of advanced head and neck cancer requires a multimodal approach with surgery and radiotherapy typically given with concurrent chemotherapy. Nearly 60% of patients are diagnosed with locally advanced but non‐metastatic disease for whom combined modality treatment is regarded as standard.^1^, ^2^ The sites of head and neck squamocellular carcinoma treated with radiotherapy are surrounded by or adjacent to bony structures that during operative treatment can require resection and subsequent reconstruction. This is equally true for advanced brain tumors, which are also often treated with the combination of surgery and post‐operative radiotherapy.^3^, ^4^, ^5^, ^6^

Surgical treatment often involves the use of foreign material in the reconstruction of both anatomy and function.^7^ The reconstructive material is thereafter present at the time of postoperative radiotherapy. The presence of foreign metallic material can cause problems for radiotherapy due to radiation scattering and absorption.^8^ The complications of radiation dose enhancement can vary from local irritation and impaired wound healing to osteoradionecrosis.^9^, ^10^ Soft tissue atrophy due to scattered radiation has also been reported as a complication when using metallic reconstructive implants.^11^ An implant material that does not impact on dose distribution of radiotherapy or interfere with diagnostic imaging such as computer tomography (CT) or magnetic resonance imaging (MRI) would be ideal. Materials used in reconstruction include autologous bone, titanium and its alloys, polyether ether ketone (PEEK) and fiber‐reinforced composites (FRC).^12^ It is important to know how these materials interact with ionizing radiation where risk for under‐ or overdosing may contribute to failure of treatment.

It is known that titanium causes radiation scattering and absorption resulting in dose enhancement or reduction in the surrounding tissue.^13^ Titanium also interferes with diagnostic imaging and causes imaging artifacts that can be problematic in postoperative follow‐up.^14^, ^15^, ^16^ There is need for a non‐metallic reconstructive material that is both durable and biocompatible. Glass fiber reinforced composites (FRC) were first introduced as a reconstructive material for dental hard‐tissue and are now also in use in craniofacial surgery.^17^, ^18^, ^19^, ^20^, ^21^, ^22^ Particles of bioactive glass (BG) have been added to FRC implants to improve osteoconductivity, osteogenicity and antimicrobial properties.^23^, ^24^, ^25^, ^26^, ^27^ Glass FRC and cortical bone have very similar radio‐opacity and therefore glass FRC does not cause artifacts in diagnostic imaging unlike metallic materials.^28^

The aim of this study was to examine the effects of the different materials used in reconstructive surgery on the surrounding environment during radiotherapy using measurements with film dosimetry and diamond detector dosimetry. The materials tested were titanium, PEEK, glass fiber reinforced composite with and without bioactive glass S53P4 and bone. In composite materials theoretical backscatter calculations are not trivial and measurements are an appropriate alternative. The hypothesis was that FRC‐BG causes less scattering and absorption than titanium and that the composite material and bone interact similarly with ionizing radiation.

## 2 MATERIALS AND METHODS

**Figure 1:**
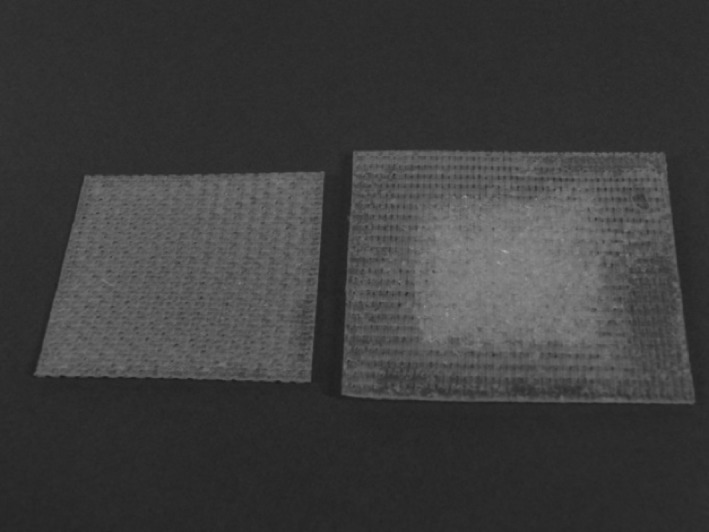
FRC (left) and FRC‐BG (right) samples. FRC, fiber‐reinforced composites.

Sandwich‐like glass FRC‐BG implant simulating specimens were used in this study. Titanium, polyether ether ketone (PEEK) and bone were used as controls. The materials used in preparation of the FRC samples are listed in Table 1. The shell of the sandwich‐like FRC‐BG implant was made of three sheets of woven glass FRC fabric of weight of 120 g/m^2^ which were impregnated with a monomer resin of 65:35 wt‐% bisphenol‐A‐glycidyldimethacrylate (BisGMA) – triethylene glycoldimethacrylate (TEGDMA) including a photosensitive initiator system containing 0.8% camphorquinone and 0.8% N,N‐dimethylaminoethyl methacrylate (DMAEMA). Resin impregnation left mesh‐like holes in the specimen surface, which in clinical conditions allow blood absorption to the implant to occur.^29^ One sheet of the FRC fabric was used on the outer surface of the FRC implant and the other two sheets on the inner surface. The space between the outer and inner surfaces was filled with particles of bioactive glass (S53P4, particle size: 500‐1000 μm, BonAlive Biomaterials, Turku, Finland). The total weight fraction of glass particles in the FRC implant was 35 w‐%. The FRC fabric was joined together and sealed the area that stretched 3 mm from the edge of the FRC specimen. The design of the FRC and FRC‐BG specimen is shown in Fig. 1. The final thickness of the sandwich‐like FRC specimens was 1.0 mm at the margins where the outer and inner implant surface joined together, and 1.5 mm in the areas containing glass particles. The resin matrix was polymerized with 3M Espe Elipar S10 photocurer for 2 × 20 s, vacuum photocuring unit 3M Espe VisioBeta Vario for 15 min and light furnace with temperature increase up to 90°C (Ivoclar Vivadent Lumamat 100) for 25 min.

**Table 1:** Materials used in the FRC and FRC‐BG samples.

Brand	Manufacturer	Lot.
Bis‐GMA	Esstech Europe ltd	751‐42
TEGDMA	Sigma‐Aldrich Co. LCC	STBC5193V
CQ	Sigma‐Aldrich Co. LCC	STBC7007V
DMAEMA	Sigma‐Aldrich Co. LCC	1437599V
120 g/m^2^ woven E‐glass fiber fabric	Ahlstrom Glassfibre Oy	NA
BonAlive glass granules	Vivoxid ltd	BG‐11/08, BG‐03/09

Bis‐GMA, bisphenol‐A‐glycidyldimethacrylate; CQ, camphorquinone; DMAEMA, N,N‐dimethylaminoethyl methacrylate; FRC, fibre‐reinforced composites; TEGDMA, triethylene glycoldimethacrylate.

Control specimens were made of commercially pure titanium sheet (Grade 2, Permascand Ljungaverk, Sweden, Lot B26589). Thickness of the sheet was 1.0 mm and several sheets were layered for having thicker specimens. Polyether ether ketone (PEEK; Mectalent Ltd, Oulu, Finland) specimens were sawn to the thickness of 1.0 mm and 3.8 mm.

The attenuation and back scatter caused by the implant materials was studied under high energy radiation fields. The measurements were performed using a linear accelerator (TrueBeam, Varian Medical Systems, Inc., Palo Alto, CA, USA) with two different energies (6 MV and 10 MV photon beams). All the measurements were done at the central axis of the radiation field, and the samples were perpendicular to the radiation field.

### 2.1 Film dosimetry

**Figure 2:**
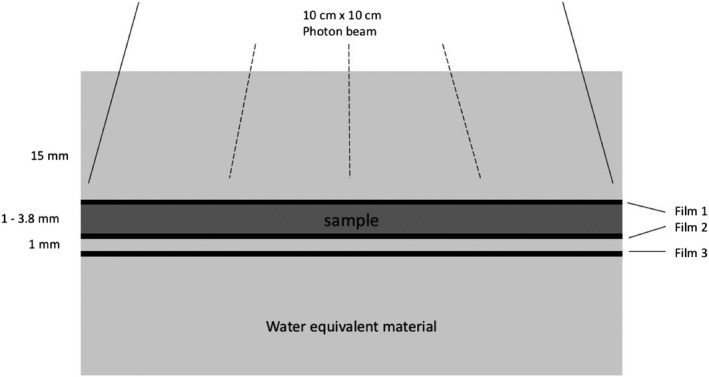
The setup of film dosimetry measurements.

The absorption and scattering of the radiation in studied materials were measured using a Gafchromic EBT3 film (Ashland ISP, Wayne, NJ, USA) in a water equivalent solid material. Three films were placed near the specimen plates. One in contact with the plates above them, giving the information about backscattering, and two other films behind the plates, one in contact and one at 1.5 mm distance giving information about the scattering and absorption (Fig. 2). The films were exposed at the dose level of 2 Gy. Since the EBT3 film is a radiochromic film, it has self‐developing feature, and therefore no chemical, thermal or physical processing was needed. Films were scanned with a flatbed scanner (Epson Perfection V700 scanner, Seiko Epson Corporation, Tokyo, Japan), and analyzed with OmniPro I’mRT software (IBA Dosimetry GmbH, Germany). The dose values were determined by calculating the mean value within a 1.5 cm^2^ ROI in the center of the sample plate.

### 2.2 Diamond detector dosimetry

**Figure 3:**
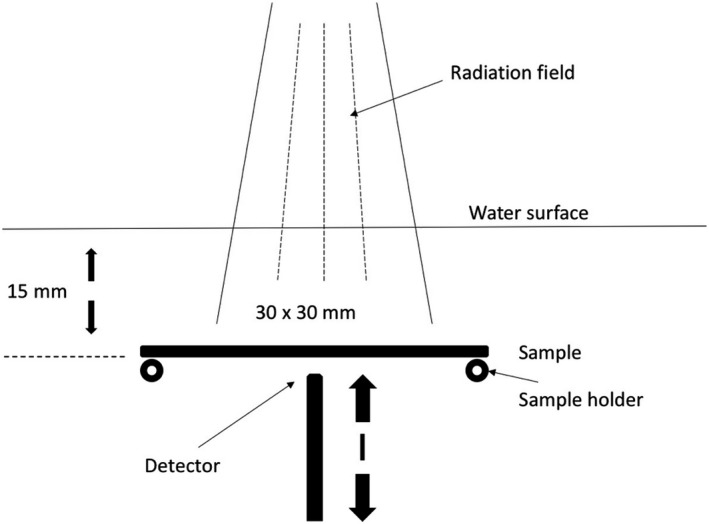
The setup of diamond detector measurements.

The radiation dose behind the sample material was measured using a single crystal diamond detector (microDiamond 60019, PTW Freiburg GmbH, Germany). The microDiamond measurements were made in a water phantom (BluePhantom2, IBA Dosimetry GmbH, Germany). The detector was mounted in a vertical orientation (Fig. 3) and the dose was measured in seven different points (1–20 mm) behind the sample material.

## 3 RESULTS

### 3.1 Film dosimetry

The results are shown in Table 2. The dose distribution in the water equivalent material was measured without the samples to serve as control values. Measurements with the samples showed that the FRC‐BG did not cause measurable dose enhancement on top of the sample due to backscattering. The backscattering with the FRC measured on top of the implant resulted in a dose enhancement of 4–6% depending on the radiation energy. With PEEK the dose enhancement on top of the material was 10% with 6 MV and 7% with 10 MV energy. Titanium caused the most backscattering with a dose enhancement of 18%. Titanium also caused the most absorption resulting in a dose reduction of 15% with 6 MV energy immediately behind the sample.

**Table 2:** Results of the film dosimetry (relative to water equivalent material alone).

Sample	6 MV			10 MV		
	Film 1	Film 2	Film 3	Film 1	Film 2	Film 3
FRC‐BG (3 mm)	0%	0%	0%	0%	0%	0%
FRC (3 mm)	4%	1%	0%	6%	0%	0%
PEEK (3.8 mm)	10%	0%	−1%	7%	1%	1%
Ti (3 mm)	18%	−15%	−9%	17%	−5%	−4%

FRC‐BG, glass fiber reinforced composite with bioactive glass; FRC, glass fiber reinforced composite; PEEK, polyether ether ketone; Ti, titanium.

### 3.2 Diamond detector dosimetry

**Figure 4:**
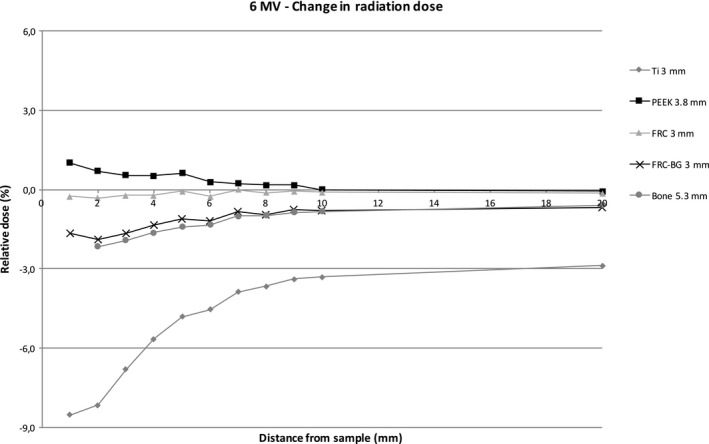
Change in radiation dose with increasing distance with 6 MV energy.

**Figure 5:**
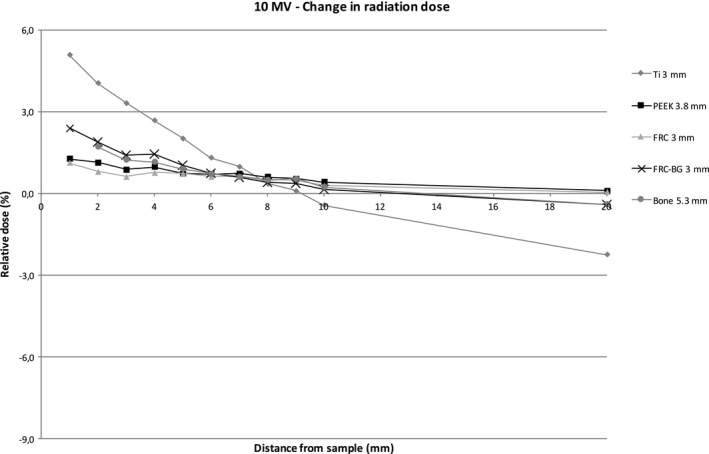
Change in radiation dose with increasing distance with 10 MV energy.

Figures 4 and 5 show the relative dose of radiation at an increasing distance with different sample materials compared to measurements without the sample. The sample thicknesses were similar for better comparison. With the 6 MV energy, titanium caused a dose reduction of 8.5% at 1 mm behind the sample with the 3 mm thick titanium sample. The difference decreases quickly with growing distance due to scattering. PEEK causes a maximum dose enhancement of 1.1% at 1 mm distance. With FRC, FRC‐BG, and bone the changes in radiation dose are minimal. With 10 MV energy scattering causes an increase resulting in a small dose enhancement with all materials. The effect is strongest with titanium with a maximum of 5.1% at 1 mm distance. The other sample materials cause less scattering resulting in a maximum change of 2.4% at 1 mm distance with the FRC‐BG.

**Figure 6:**
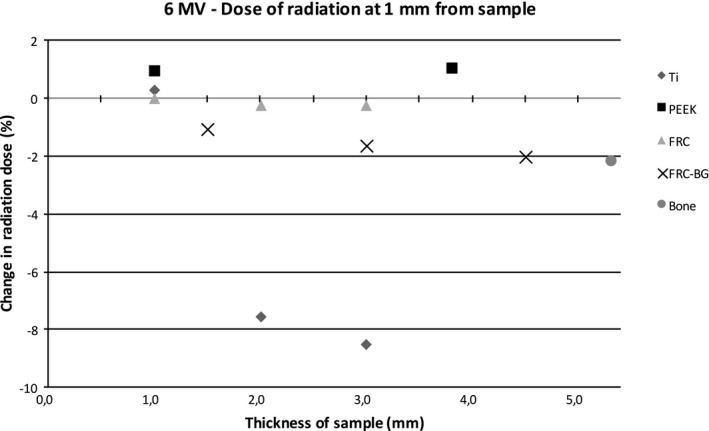
Change in dose of radiation with 6 MV energy measured with the diamond detector with different sample thicknesses at 1 mm distance.

**Figure 7:**
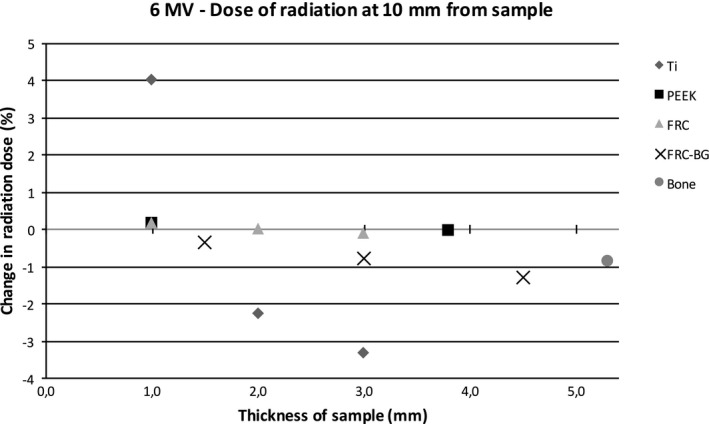
Change in dose of radiation with 6 MV energy measured with the diamond detector with different sample thicknesses at 10 mm distance.

**Figure 8:**
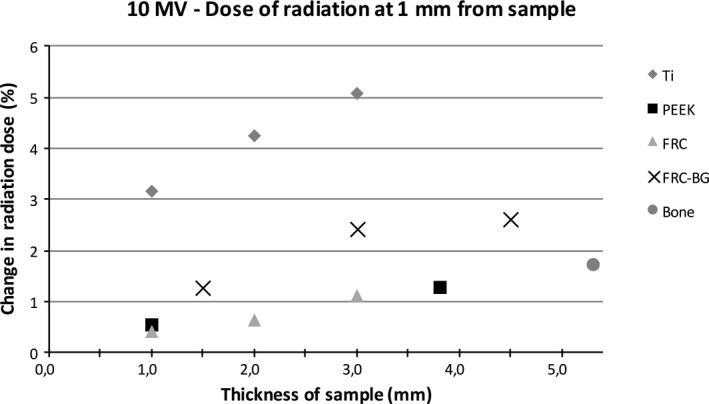
Change in dose of radiation with 10 MV energy measured with the diamond detector with different sample thicknesses at 1 mm distance.

**Figure 9:**
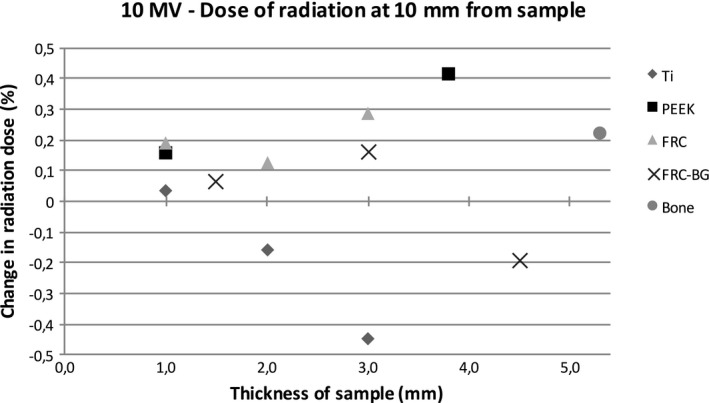
Change in dose of radiation with 10 MV energy measured with the diamond detector with different sample thicknesses at 10 mm distance.

Figures 6, 7, 8, 9 show the effect of different material thicknesses to the radiation dose at the distances of 1 mm and 10 mm. At 1 mm behind the sample with 6 MV radiation, titanium had the most effect on radiation dose when compared to the other materials. A minimal dose enhancement was caused by 1 mm titanium plate due to scattering. With the 2 mm and 3 mm samples the dose reduced 7.6% and 8.5%, respectively. The other materials with different thicknesses including bone at 5.3 mm caused a maximum of 2% difference in radiation dose. At 10 mm distance, titanium caused a dose enhancement of 4% with a 1 mm sample and a dose reduction of 2–3% with thicker samples. FRC, FRC‐BG, PEEK, and bone all caused a change in radiation dose of up to 1.3%. With 10 MV energy, an increase in radiation dose was measured at 1 mm distance with all materials. The change was largest for the 3 mm titanium plate with a 5.1% dose enhancement. With FRC‐BG a maximum enhancement of 2.6% was measured. The change in dose fell close to zero rapidly due to scattering.

## 4 DISCUSSION

The effects of bone reconstruction materials on photon radiation were measured for 5 different materials. Main interest was in the effects of the novel composite material compared especially to titanium which is the gold standard in bone reconstruction.

The thickness and required strength of the reconstruction material used in head and neck surgery vary with site and function. Load‐bearing sites such as the mandible are subject to much greater forces than, for example, the calvarium. Therefore, the requirements for reconstructive materials vary a lot and depend on the site of implantation. In addition, the size of the reconstructed area sets certain requirements for the reconstruction material. In cranial bone surgery, large defects are possible and require sufficient coverage by the implant. This in turn increases the area in risk for radiation‐induced complications with materials that cause significant scattering.

The dominant interaction of high energy photons with materials is Compton scattering and the effect increases with material electron density (EDV, number of electrons per cm^3^) while remaining independent of effective atomic number (Z).^30^ As EDV = Electron density per gram (EDG, number of electrons per gram) x physical density (PD, g/cm^3^), it has been shown that the backscatter dose increases with EDV and thus with PD.^31^, ^32^ Usually high Z material has high physical density but this is not a consistent phenomenon. The physical density of pure titanium is 4.50 g/cm^3^, PEEK 1.32 g/cm^3^, FRC 1.20 g/cm^3^, bioactive glass 2.6 g/cm^3^ and bone 1.85 g/cm^3^ while the density for soft tissue is 1.00 g/cm^3^. The Z for PEEK is lower than the other tested composites but the attenuation and back scatter results with tested radiation energies were similar.^33^, ^34^, ^35^ The high density of titanium in comparison with the other tested materials accounts for the higher backscattering dose.

The radiation dose increases most on top of the implant and the effect of metallic reconstruction materials on radiation is inevitable even with careful planning.^36^ The scattering caused by titanium plates during radiotherapy is greater with thicker plates than with smaller and thinner ones. Both the dose build‐up on top of the material and the dose reduction behind the material are greater as the size of the implant increases.

Our results show that different implantable reconstructive materials used in head and neck surgery and neurosurgery affect the radiation dose in the surroundings of the implanted material. Sakamoto et al.^36^, ^37^ studied the effect of different implants used in cranial surgery. The radiation dose increased most due to scattering with titanium and with growing radiation energy which is in accordance with our results. The amount of backscatter radiation was smaller with hydroxyapatite and resorbable plates. Our results are consistent with the results of Ozen et al.^38^, who measured a rise of 18% in radiation dose using 6 MV energy with a titanium dental implant. In our measurements, all of the materials except for FRC‐BG caused dose enhancement on top of the material. The enhancement was highest with titanium, which increases the risk of damage to the overlying tissue the most. The results are also in line with previous studies measuring the effects of titanium on radiation.^13^, ^39^, ^40^

In our measurements the glass fiber reinforced composite did not cause marked dose enhancement or reduction. The changes in radiation dose were greatest with titanium. The use of a composite reconstruction material could thus provide the surgical site a better environment for healing during and after radiotherapy.

The results with the novel material are promising considering a reconstructive material that has proven to work in cranial reconstruction^22^ and causes less imaging artifacts in CT and MRI compared to titanium.^41^, ^42^ The development of nonmetallic implants for craniofacial bone reconstruction has led to the introduction of implants made of composite materials.^43^ Composite implants allow radiotherapy but in order to be detectable in CT and MRI they need radio‐opacifying filling material which in turn can cause imaging artefacts.^28^ Glass fiber reinforced composite has the advantage of being radio‐opaque and allowing radiotherapy without dose enhancement.

It is important to understand the effects of the reconstruction material on radiation dose both on the entrance and on the exit side of the implant. The effects can have an impact on treatment outcome and the healing of the operated area. Increase in the radiation dose in the bone and soft tissue adjacent to the implant material can contribute to difficulties in healing.^44^ There is no specific amount of radiation that is known to cause osteoradionecrosis but the risk is shown to increase with dose.^45^ Our results show that the dose reduces most with titanium compared to other tested reconstruction materials. The possibility to provide a better healing environment is promising. Fiber‐reinforced composite does not cause radiation scattering to a measurable account and seems in that perspective to be a good alternative to titanium.

A limitation to the study is that the results are not directly comparable to a clinical setting. The study setting was kept simple in order to eliminate measurement artifacts thus making it easier to understand the differences between the tested materials. Single direct photon beams are not typically used in radiation therapy anymore, instead almost all curative intent head and neck radiation treatments are delivered with multiple IMRT beams or dynamically rotating fields (VMAT). However we considered it a good way of comparing the performance of the materials. Assessing the clinical effect of each material separately with a modern radiation treatment plan, using an anthropomorphic phantom and measurements or Monte Carlo simulation, would make it harder to compare the differences between the materials since the plan would vary with different materials present.

We conclude that glass fiber reinforced composite is a promising material based on its minimal interaction with photon radiation. We found that this material is safer than titanium in terms of tissue healing and predictability of dose distribution during radiotherapy. Glass fiber reinforced composite may be a material of choice for craniofacial surgery for patients undergoing multimodal treatment of head and neck cancer.

## CONFLICT OF INTEREST

Author Pekka Vallittu is a member of the Board and shareholder of Skulle Implants Corporation.
